# Co-expression networks in generation of induced pluripotent stem cells

**DOI:** 10.1242/bio.016402

**Published:** 2016-02-18

**Authors:** Sharan Paul, Lance Pflieger, Warunee Dansithong, Karla P. Figueroa, Fuying Gao, Giovanni Coppola, Stefan M. Pulst

**Affiliations:** 1 Department of Neurology, University of Utah, Salt Lake City, UT 84132, USA; 2 Department of Biomedical Informatics, University of Utah, Salt Lake City, UT 84108, USA; 3 Departments of Psychiatry and Neurology, Semel Institute for Neuroscience and Human Behavior, David Geffen School of Medicine, University of California Los Angeles, Los Angeles, CA 90095, USA

**Keywords:** Adenoviral delivery, Gene expression, Reprogramming, iPSC

## Abstract

We developed an adenoviral vector, in which Yamanaka's four reprogramming factors (RFs) were controlled by individual CMV promoters in a single cassette (Ad-SOcMK). This permitted coordinated expression of RFs (SOX2, OCT3/4, c-MYC and KLF4) in a cell for a transient period of time, synchronizing the reprogramming process with the majority of transduced cells assuming induced pluripotent stem cell (iPSC)-like characteristics as early as three days post-transduction. These reprogrammed cells resembled human embryonic stem cells (ESCs) with regard to morphology, biomarker expression, and could be differentiated into cells of the germ layers *in vitro* and *in vivo*. These iPSC-like cells, however, failed to expand into larger iPSC colonies. The short and synchronized reprogramming process allowed us to study global transcription changes within short time intervals. Weighted gene co-expression network analysis (WGCNA) identified sixteen large gene co-expression modules, each including members of gene ontology categories involved in cell differentiation and development. In particular, the brown module contained a significant number of ESC marker genes, whereas the turquoise module contained cell-cycle-related genes that were downregulated in contrast to upregulation in human ESCs. Strong coordinated expression of all four RFs via adenoviral transduction may constrain stochastic processes and lead to silencing of genes important for cellular proliferation.

## Abstract (precis)

**Summary:** We developed a novel adenoviral iPSC reprogramming vector integrating Yamanaka's four factors in a single cassette, allowing for the identification of biologically relevant co-expression networks.

## INTRODUCTION

Stem cell research offers great promise for understanding basic mechanisms of human cellular development and differentiation. Stem cells and their differentiated products have significant therapeutic potential for treatment of human diseases. Yamanaka revolutionized the stem cell research field by using reprograming factors (RFs) to reprogram somatic cells into an embryonic stem cell (ESC)-like state, known as induced pluripotent stem cells (iPSCs) (Takahashi and Yamanaka, 2006). This technology bypasses ethical concerns and can produce stem cells from an individual that are compatible with his/her own immune system.

Mouse and human somatic cells can be reprogrammed by delivering four RFs (OCT3/4, SOX2, KLF4 and c-MYC; or OCT3/4, SOX2, NANOG and LIN28) (Takahashi et al., 2007; Okita et al., 2007; Wernig et al., 2007; Yu et al., 2007; Park et al., 2008) via simultaneous lenti- or retroviral transduction. This technology has major limitations because the lenti- or retroviral based gene delivery systems result in permanent integration of the transgene and/or vector sequences into the genome. These lasting events cause residual transgene expression resulting in low reprogramming efficiency and slow kinetics. To overcome these problems, several non-viral approaches have been used such as transient transfection, RNA transfection, the ‘piggyback’ system, protein transduction, the Cre-LoxP excision system, minicircle vectors, episomal plasmids, non-integrating adenoviral vectors and Sendai virus (Kim et al., 2009; Woltjen et al., 2009; Okita et al., 2008; Zhou et al., 2009; Jia et al., 2010; Warren et al., 2010; Stadtfeld et al., 2008; Zhou and Freed, 2009; Ban et al., 2011). Nevertheless, limitations related to reprogramming efficiency and complexity of genetic manipulation still persist in reprogramming. Improved reprogramming efficiency has been achieved to some extent by polycistronic lentiviral vector system where RFs are connected in-frame via self-cleaving 2A peptides or IRES sequences (Shao et al., 2009; Sommer et al., 2009; Chang et al., 2009; Carey et al., 2009). However, inefficient polypeptide cleavage in transduced cells or low translation efficiency of polycistronic RNAs may limit this approach.

Even though iPSCs share similar properties with human ESCs including the maintenance of the stem cell state and the potential for differentiation, the degree of molecular similarity between iPSCs and ESCs has not been completely elucidated (Bock et al., 2011). Much progress has been made in recent years to identify the critical role of gene expression changes in the induction and maintenance of pluripotency. Many studies suggest that iPSCs are “nearly identical” to their embryo-derived counterparts, but the biological significance of the small percentage of genes that are differentially expressed between iPSCs and ESCs remains unclear. As the methods used to reprogram somatic cells into iPSCs vary, the resulting iPSCs may differ at the cellular or molecular level, including genomic integrity, epigenetic stability, as well as expression of noncoding and coding RNAs. To date, no reports have described the full extent of molecular changes that occur over the course of reprogramming.

Nevertheless, with recent advances in the iPSCs field, a large amount of knowledge has been gained relating to the characterization of iPSCs. Reprogramming of somatic cells is associated with multiple-step processes mediated by gene expression changes that progressively induce the expression of ESC-like genes and suppress the somatic cell genetic program (Brambrink et al., 2008). Gene expression profiling in fibroblasts revealed three phases of reprogramming termed as initiation, maturation, and stabilization, with the initiation phase primarily marked by a mesenchymal-to-epithelial transition (MET) (Li et al., 2010; Samavarchi-Tehrani et al., 2010). Recent evidence suggests that the reprogramming factors initiate a sequence of probabilistic events that eventually lead to acquisition of small and unpredictable fraction of iPSCs (Hanna et al., 2009; Yamanaka, 2009). Because these studies were based on the analysis of heterogeneous cell populations at different stages of the reprogramming process, it has been difficult to clarify reprogramming kinetics and functional relevance of transcriptomic changes during reprogramming.

To achieve uniform reprogramming within a homogenous culture, we have generated an adenoviral vector in which RFs are controlled by individual CMV promoters in a single cassette, designated as Ad-SOcMK. Transduction of fibroblasts with the Ad-SOcMK vector resulted in synchronized changes in a relatively short period of time. The resulting reprogrammed cells demonstrated an ESC-like phenotype with regard to morphology and pluripotency marker expression but failed to achieve self-renewal activity. As synchronization of cells was seen in such a short period of time with the adenoviral delivery system, we studied global gene expression changes during reprogramming and determined the correlation between gene expression changes and re-programming that might explain the lack of self-renewal activity of reprogrammed cells.

## RESULTS

### Reprogramming of human IMR90 cells and initial characterization

**Fig. 1.**
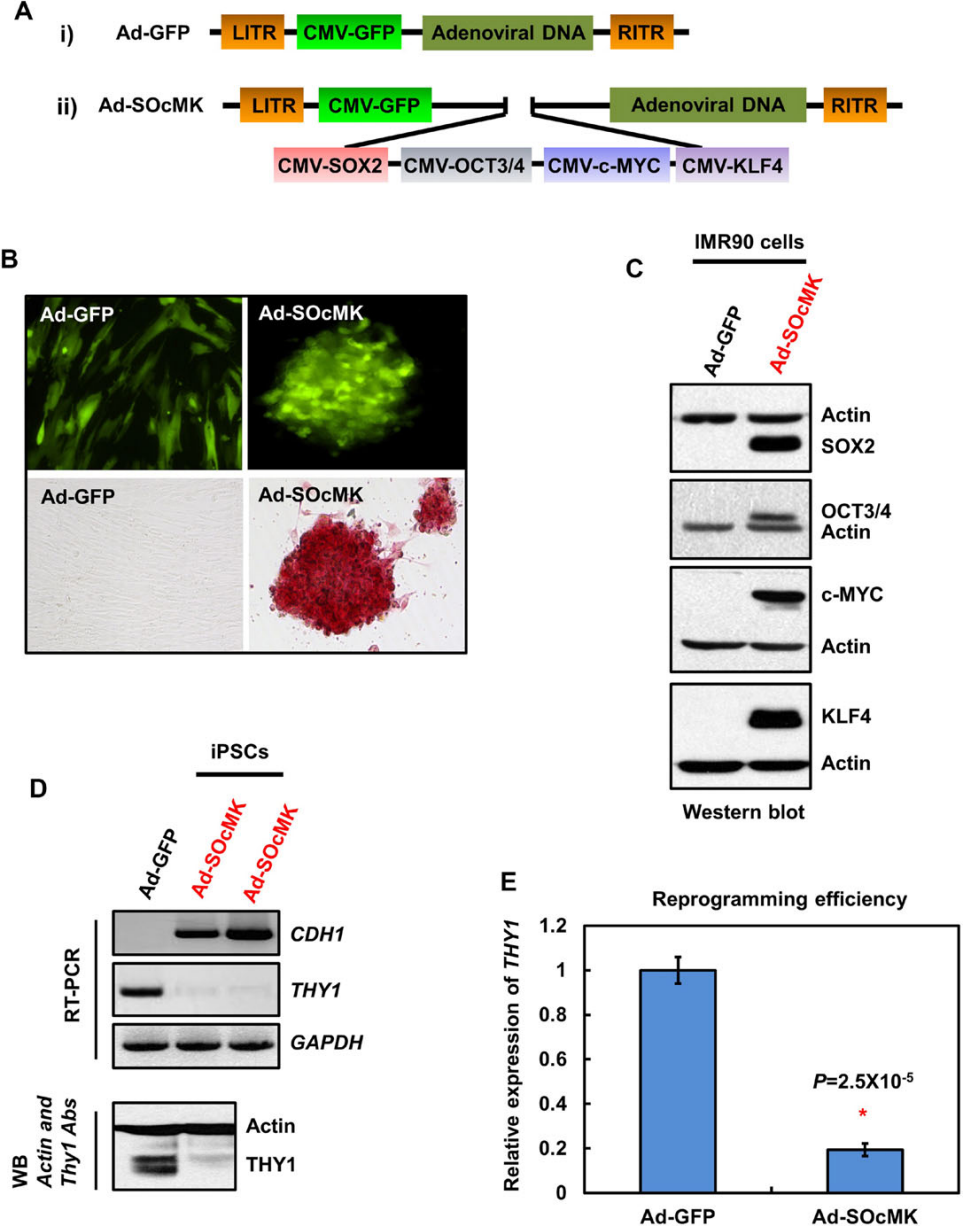
**Reprogramming of somatic cells with adenovirus containing reprogramming factors (RFs) in a single cassette.** (A) Schematic representations of adenovirus-GFP (Ad-GFP) (i), and adenoviral vector containing RFs (Ad-SOcMK) (ii). RFs SOX2, OCT3/4, c-MYC and KLF4 are controlled individually by separate CMV promoters in a single cassette. (B) Transduction of human IMR90 cells with Ad-SOcMK, but not with Ad-GFP, results in the appearance of cell clumps by days 2-3. Photomicrographs of Ad-GFP- and Ad-SOcMK-transduced cells with GFP expression (upper panel) on day 3 post-transduction. Lower panel: alkaline phosphatase (ALP) cytochemistry assay on day 3. IMR90 cells transduced with Ad-SOcMK stain for ALP on day 3, in contrast to IMR90 cells transduced with Ad-GFP. Representative micrographs of three independent experiments are shown. All images at 10× magnification. (C) IMR90 cells transduced with Ad-SOcMK express exogenous RFs. Protein extracts from the harvested IMR90 cells transduced with Ad-GFP or Ad-SOcMK on day 3 were subjected to western blot analyses. The blots were re-probed for actin as an internal loading control. The blot represents one of three independent experiments. (D) Switch in transcript (upper panel) and protein expression (lower panel) of two key genes involved in the mesenchymal to epithelial transition (MET). *THY1*, but not *CDH1* is expressed in IMR90 cells. On day 3 post-transduction with Ad-SOcMK, strong expression of *CDH1* is seen, whereas *THY1* expression is suppressed. For RT-PCR, *GAPDH* is used as internal control, for western blot analysis, actin serves as loading control. (E) Quantification of reprogramming efficiency of IMR90 cells. Synthesized cDNAs were subjected to qPCR analysis to measure the expression levels of *THY1* mRNA in Ad-SOcMK-transduced cells at day 3, with *GAPDH* mRNA as an internal control. Error bars (±) represent standard deviation (*n*=3). *P*-values were determined by paired Student's *t*-test.

In an effort to avoid uneven gene delivery and/or inefficient cleavage of polycistronic RNAs or polypeptides in single-vector approach, we developed an adenoviral vector in which RFs (**S**OX2, **O**CT3/4, **c-M**YC and **K**LF4) were controlled by individual CMV promoters in a single cassette, designated as Ad-SOcMK. In addition, a CMV-driven GFP marker was placed separately in the vector to monitor transduction efficiency during reprogramming. We validated the expression of individual protein factors in SH-SY5Y cells (Fig. S1) before proceeding to generate the adenovirus (Fig. 1A). Next, we used the Ad-SOcMK virus reprogramming system to test the ability to reprogram IMR90 cells. In brief, IMR90 cells were transduced with Ad-SOcMK or Ad-GFP for 12 or 24 h after which time the medium was replaced with human ES cell medium. Within 1 day, Ad-SOcMK-transduced cells took on a different appearance and began to form small cell clusters. By day 2 or 3, several colonies of cells showing ES cell-like morphology emerged in the dish (Fig. 1B). Alkaline phosphatase (ALP) assays revealed strong staining of reprogrammed cells consistent with pluripotency (Fig. 1B). Western blot analyses of harvested cells demonstrated expression of all RFs in Ad-SOcMK-transduced cells (Fig. 1C).

### Mesenchymal-to-epithelial transition (MET)

**Fig. 2.**
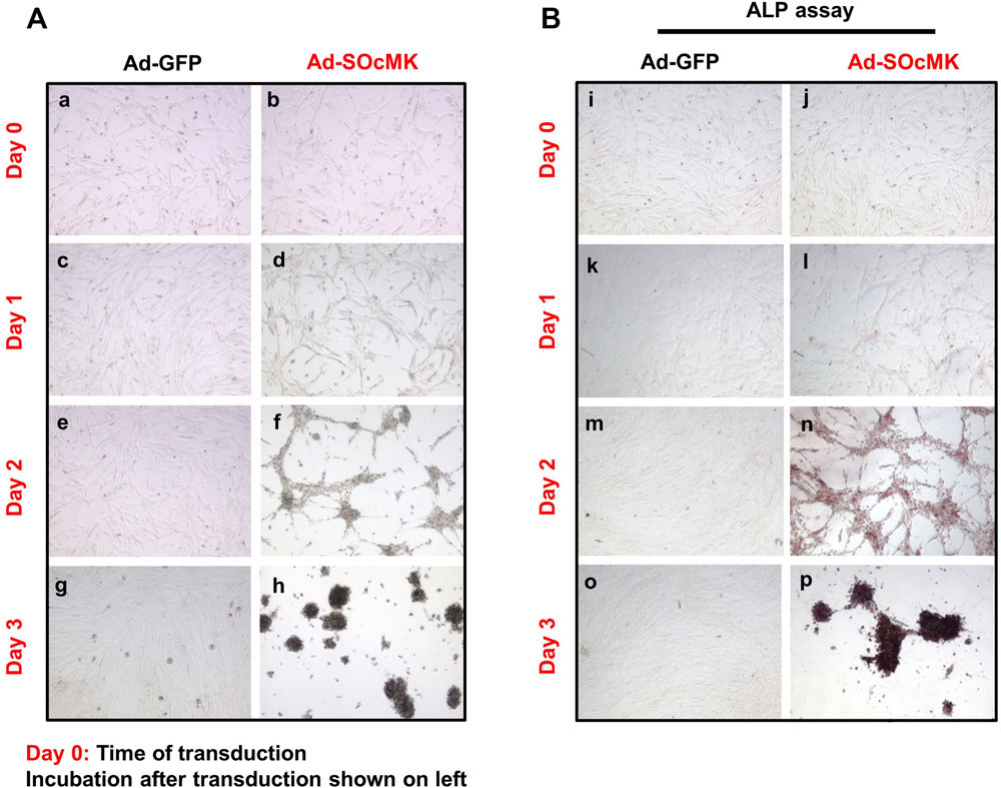
**Rapid cellular changes in IMR90 cells after transduction with Ad-SOcMK.** Alterations of morphology (Ab,d,f,h) and ALP expression (Bj,l,n,p) of Ad-SOcMK-transduced IMR90 cells with time after transduction are shown. Within one day, Ad-SOcMK-transduced cells show a different morphology (Ad) than Ad-GFP-transduced cells (Ac) with clear clustering (Af) and ALP expression by day 2 (Bn). In Ad-GFP-transduced cells, alterations of cell morphology (Aa,c,e,g) or ALP expression (Bi,k,m,o) are not seen. Ad-GFP or Ad-SOcMK adenoviruses were removed after one day (designated day 1), replaced with human ESC medium, and cell morphology was monitored. All phase contrast photomicrographs (A) and ALP cytochemistry images (B) were taken at 4× magnification. Representative micrographs of three independent experiments are shown.

Mesenchymal-to-epithelial transition (MET) is the key regulatory event during reprogramming of somatic cells to the pluripotent state. This event is associated with depletion of the mesenchymal marker, THY-1 cell surface antigen, and up-regulation of the epithelial marker, cadherin 1 (CDH1) (Li et al., 2010; Chen et al., 2010). To investigate this, we measured the steady-state levels of THY1 and CDH1 in Ad-SOcMK-transduced cells. RT-PCR and western blot analyses revealed up-regulation of CDH1 and concomitant reduction of THY1 in Ad-SOcMK-transduced cells when compared with control infected cells (Fig. 1D). As cells dramatically switched the state in a short period of time, we determined the expression level of *THY1* by quantitative PCR (qPCR) as a function of reprogramming efficiency. qPCR data revealed a decrease in levels of *THY1* by ∼80% in Ad-SOcMK-transduced cells (Fig. 1E). One of the crucial morphological changes during MET is the transformation of elongated fibroblasts into tightly packed clusters of rounded cells. We observed that Ad-SOcMK-transduced cells underwent progressive epithelial-like morphological changes from elongated fibroblasts (Fig. 2Ab) to packed clusters of rounded cells as visualized by phase contrast microscopy (Fig. 2Ad,f,h). Morphological changes occurred in close association with expression of ALP. ALP-positive cells appeared as early as day 1 in Ad-SOcMK-transduced cells and ALP positive cells progressively increased in number as reprogramming time increased (Fig. 2Bl,n,p). Cells transduced with Ad-GFP neither showed morphological changes (Fig. 2Ac,e,g) nor staining for ALP (Fig. 2Bk,m,o). Thus, reprogramming of IMR90 cells by Ad-SOcMK resulted in rapid and specific mesenchymal to epithelial transition with very high efficiency.

### ESC marker gene expression, *in vitro* and *in vivo* differentiation

**Fig. 3.**
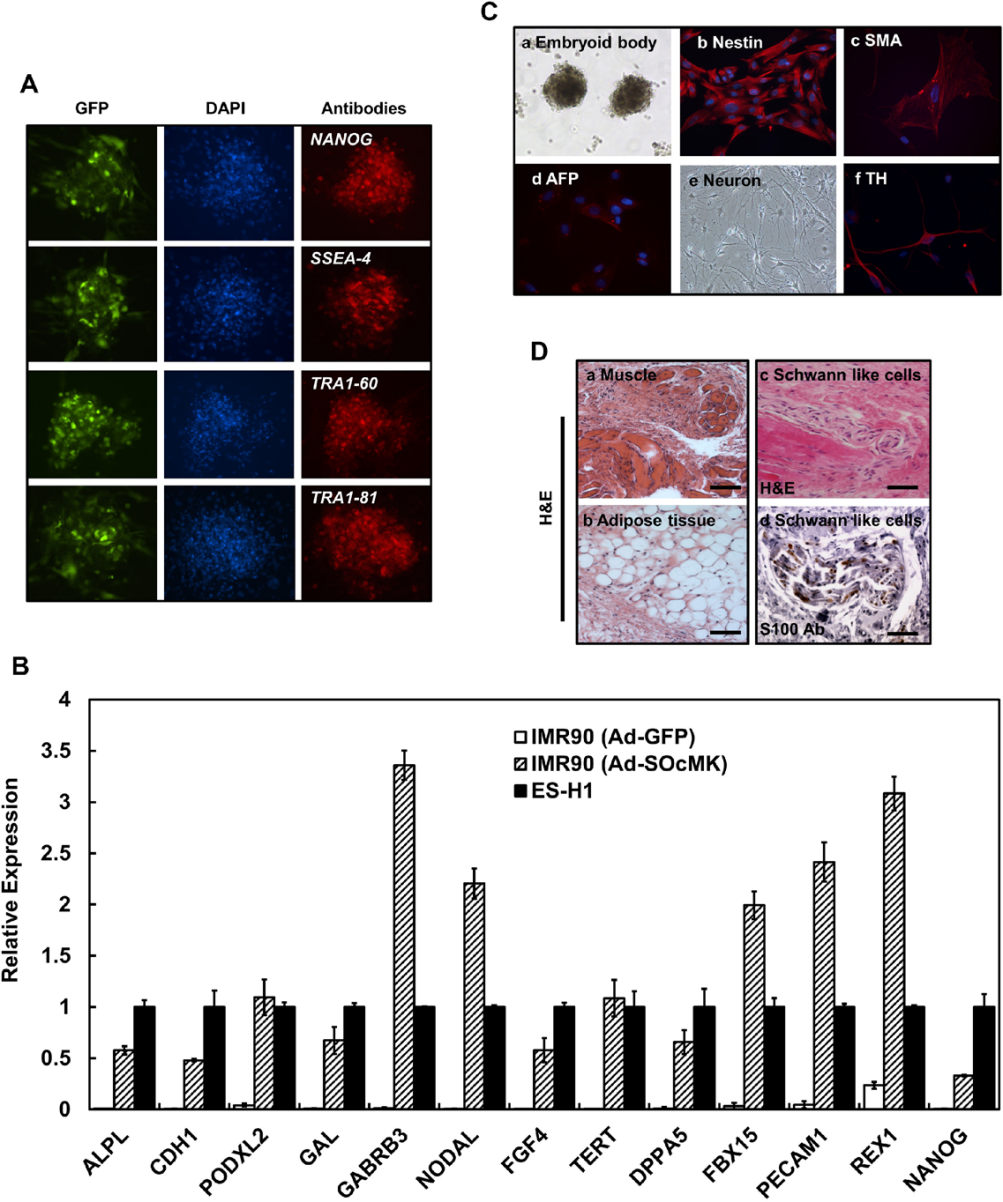
**Reprogrammed cells with Ad-SOcMK express endogenous ES cell-marker genes and show pluripotency**. (A) Reprogrammed cells with Ad-SOcMK were subjected to immunofluorescence study using antibodies against the following: NANOG, SSEA-4, TRA1-60 and TRA1-81. Left panels show expression of GFP, middle panels depict nuclear staining with DAPI. The respective antibody labeling (see Table S5) is shown in the right panels. (B) Expression of ESC marker genes by qPCR is shown. IMR90 cells were transduced with Ad-GFP or Ad-SOcMK. As cells were reprogrammed, total RNA was isolated from harvested cells and subjected to qPCR analyses to determine expression of ES cell-marker genes as indicated in graph. *GAPDH* RNA was amplified as an internal control. (C) Differentiation of Ad-SOcMK-transduced IMR90 cells. On day 3, Ad-SOcMK-transduced IMR90 cells were mechanically dissociated and cultured in ESC medium (without bFGF) in non-coated T25 flasks. EBs formed after 8-9 days, as observed by phase contrast photomicrograph (a, 4× magnification). Cells in each of the three germ layers were identified with antibodies against the following proteins (see Table S5): Nestin (b) for ectodermal progenitors, SMA (c) for mesodermal progenitors, and AFP (d) for endodermal progenitors. (e,f). After plating on MEF cells, iPSCs differentiated into neuronal cells judged by phase contrast image (e, 10× magnification) and some neurons were stained with dopaminergic marker, tyrosine hydroxylase (TH) (f). (D) Subcutaneous injection of reprogrammed cells resulted in teratoma formation in NOD/SCID mice. Differentiated tissues showing muscle and adipose cell morphology were seen (a,b) as well as Schwann-like cells (H&E stain, scale bars: 100 μm) (c), adjacent section to (d) stained with an antibody against the marker protein S100 (scale bar: 50 μm).

Immunofluorescence studies demonstrated the expression of pluripotency associated markers such as NANOG, SSEA-4, TRA-1-60 and TRA-1-81 in Ad-SOcMK induced reprogrammed cells (Fig. 3A). qPCR analysis of isolated RNAs from Ad-SOcMK induced reprogrammed cells demonstrated expression of undifferentiated ES cell-marker genes, including *ALPL*, *CDH1*, *PODXL2* (podocalyxin-like 2), *GAL* (galanin prepropeptide), *GABRB3* (gamma-aminobutyric acid receptor, beta 3), *NODAL* (Nodal homolog), *FGF4* (fibroblast growth factor 4), *TERT* (telomerase reverse transcriptase), *DPPA5* (developmental pluripotency-associated 5), *FBX15* (F-box protein 15), *PECAM1* (platelet/endothelial cell adhesion molecule 1), *REX1* (ZFP42 zinc finger protein) and *NANOG* (Fig. 3B). However, when compared to human ESCs, *NANOG* levels were found to be significantly lower in our Ad-SOcMK-transduced cells.

Bisulfite genomic sequence analysis of the *NANOG* promoter demonstrated a hypomethylated state in CpGs of Ad-SOcMK-transduced cells when compared with the highly methylated CpGs in parent IMR90 cells (Fig. S2). In order to exclude viral DNA integration into genomic DNA, we performed Southern blot analyses digesting genomic DNA from Ad-SOcMK-transduced cells with *Bam*HI and *Asc*I for KLF4 and c-MYC probes, respectively. Notably, Southern blot analyses did not detect genomic integration of the adenoviral transgene into reprogrammed cells derived from IMR90 cells (Figs S3, S4). Chromosomal G-band analyses showed that reprogrammed cells with Ad-SOcMK had a normal karyotype of 46, XX (Fig. S5).

Furthermore, to establish the capacity of cells to differentiate, embryoid bodies (EBs) were generated from freshly prepared cells reprogrammed with Ad-SOcMK (Fig. 3Ca) and allowing them to spontaneously differentiate *in vitro*. Representative immunofluorescence data revealed expression of early differentiation markers; Nestin (ectoderm, Fig. 3Cb), smooth muscle actin (SMA) (mesoderm, Fig. 3Cc), and alpha-fetoprotein (AFP) (endoderm, Fig. 3Cd) which confirm *in vitro* pluripotency abilities. To test whether reprogrammed cells with Ad-SOcMK could be differentiated into neurons, cells were seeded on inactivated MEF cells and cultured for 22-25 days. Morphological and immunostaining data revealed that reprogrammed cells were differentiated into neurons with a subpopulation of neurons staining with the dopaminergic marker, tyrosine hydroxylase (TH) (Fig. 3Ce,f). To examine developmental potential *in vivo*, reprogrammed cells generated with Ad-SOcMK were injected into NOD/SCID mice subcutaneously. After 9-10 weeks, teratomas developed and histological data revealed development of muscle, adipose tissues and Schwann-like cells (Fig. 3Da-d). Thus, reprogrammed cells generated with our protocol showed pluripotency with the potential of differentiating into germ layers *in vitro* and *in vivo*.

### Reprogramming of other human somatic cells with adenoviral vector

Next, we asked whether the Ad-SOcMK virus could be applied to reprogram other human somatic cells. To test this we used human skeletal muscle cells (SkMCs), and spinocerebellar ataxia type 2 (SCA2) patient skin fibroblasts. Methods for cell culture and reprogramming are detailed in the materials and methods section. When SkMCs and SCA2 skin fibroblasts were transduced with Ad-SOcMK, several cell clumps like ES cell colonies emerged in the dishes as early as day three. The SkMC and SCA2 skin fibroblast-derived reprogrammed cells were positively stained for ALP. Immunofluorescence and RT-PCR analysis data revealed that these reprogrammed cells expressed markers associated with pluripotency and followed the MET process (Figs S6, S7). These findings demonstrate that the single cassette Ad-SOcMK virus can be used to reprogram efficiently from a number of somatic cells in a short period of time.

### Clonal expansion of reprogrammed cells

We have demonstrated the ability of human somatic cells to be reprogrammed transiently by Ad-SOcMK virus (Figs 1–3). The reprogrammed cells resembled human ESCs with regard to morphology, biomarker expression, and could be differentiated into cells of the germ layers in some extent. Thereafter, we repeatedly tried to expand the cells on culture dishes in order to assess their true pluripotency state. Surprisingly, adenovirus-mediated reprogrammed cells were unable to maintain as stable clones under ES cell culture conditions either by picking individual colonies or by passaging all cells on plates that contained multiple colonies. These unsuccessful efforts employed a variety of growth conditions including those (mouse embryo feeder layers, conditioned media, E8 media, Geltrex matrix- coated culture dishes) routinely used to establish stable iPSCs. This observation predicts perturbation of coordinated expression of transcripts during the course of reprogramming with adenovirus that hinders acquiring true stemness.

### Global gene expression changes

**Fig. 4.**
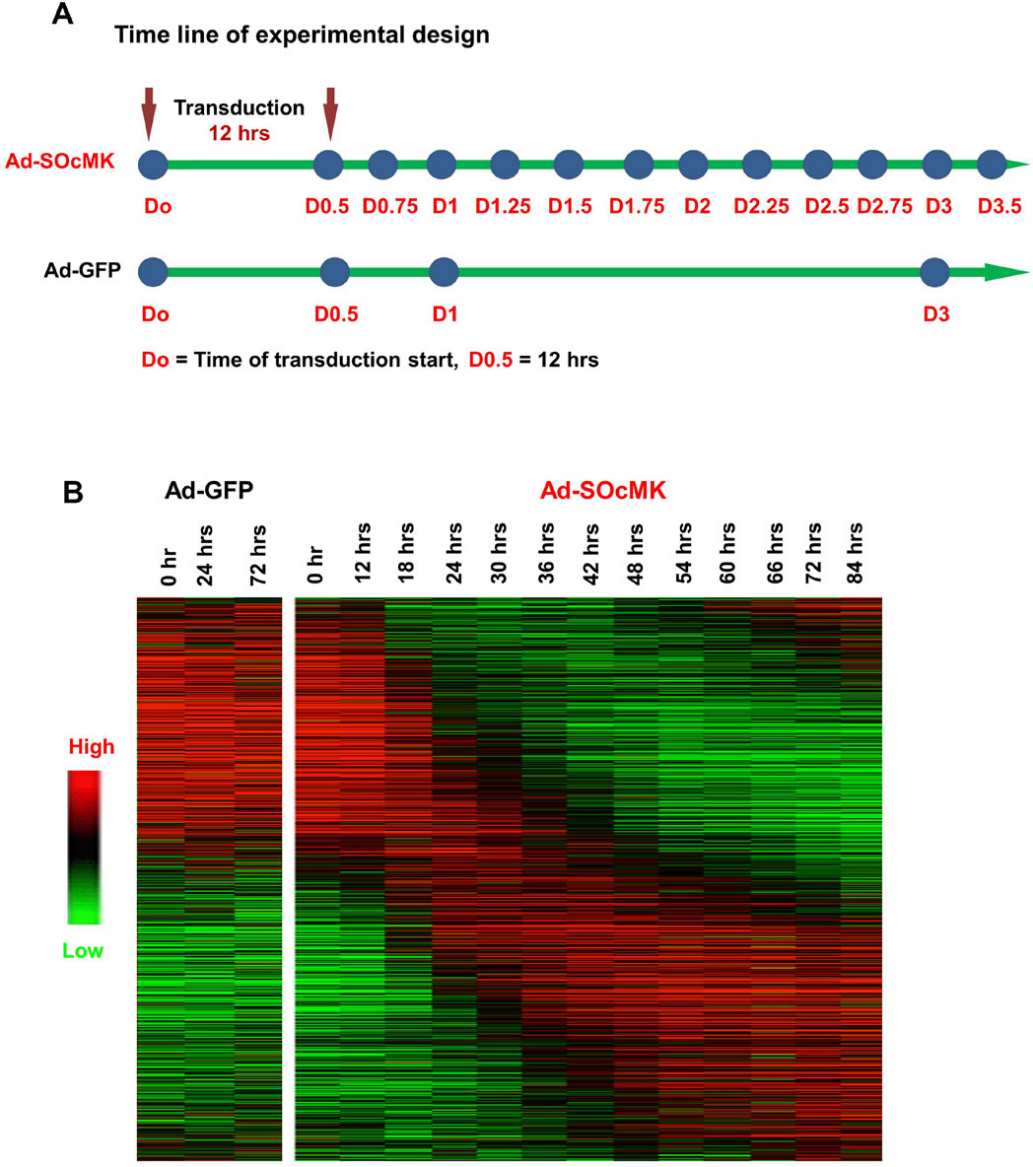
**Expression profiling reveals wave of expression changes across time points.** (A) Time line of gene expression analyses. (B) Heat-map representation of differentially expressed genes across time. Data were sorted according to the most significant eigenes (see Materials and Methods). Ad-GFP-transduced cells (control) are shown on left side of heat-map. Red, increased expression; green, decreased expression.

We observed that adenovirus-mediated reprogrammed cells were unable to maintain as stable clones under ES cell culture conditions. It was unclear whether reprogramming over a short time period to induce large scale transcriptional changes might negatively affect the propagation of reprogrammed cells. To assess the transcriptional changes, we performed two time-series experiments using expression array profiling in IMR90 cells. We isolated RNA at 0, 24, 48 and 72 h after Ad-SOcMK transduction for the first experiment and at 6 h intervals from 0 to 84 h after Ad-SOcMK transduction of IMR90 cells for the second experiment and used microarray chips to query the expression profiles of 27,958 genes and 7419 LincRNA targets (Fig. 4). The heat-map of the gene expression profile (21,372 genes, Fig. 4B) showed a large class of RNAs that were highly expressed in IMR90 cells with rapid reduction in the 12-24 h timeframe. A second class of RNAs showed a small amount of change initially, and later exhibit increased expression with a return to or below initial levels by 72-84 h. Finally, genes in a third class had low expression in the first 24-48 h but become highly expressed for the remainder of the time-series.

### Gene co-expression in modules

**Fig. 5.**
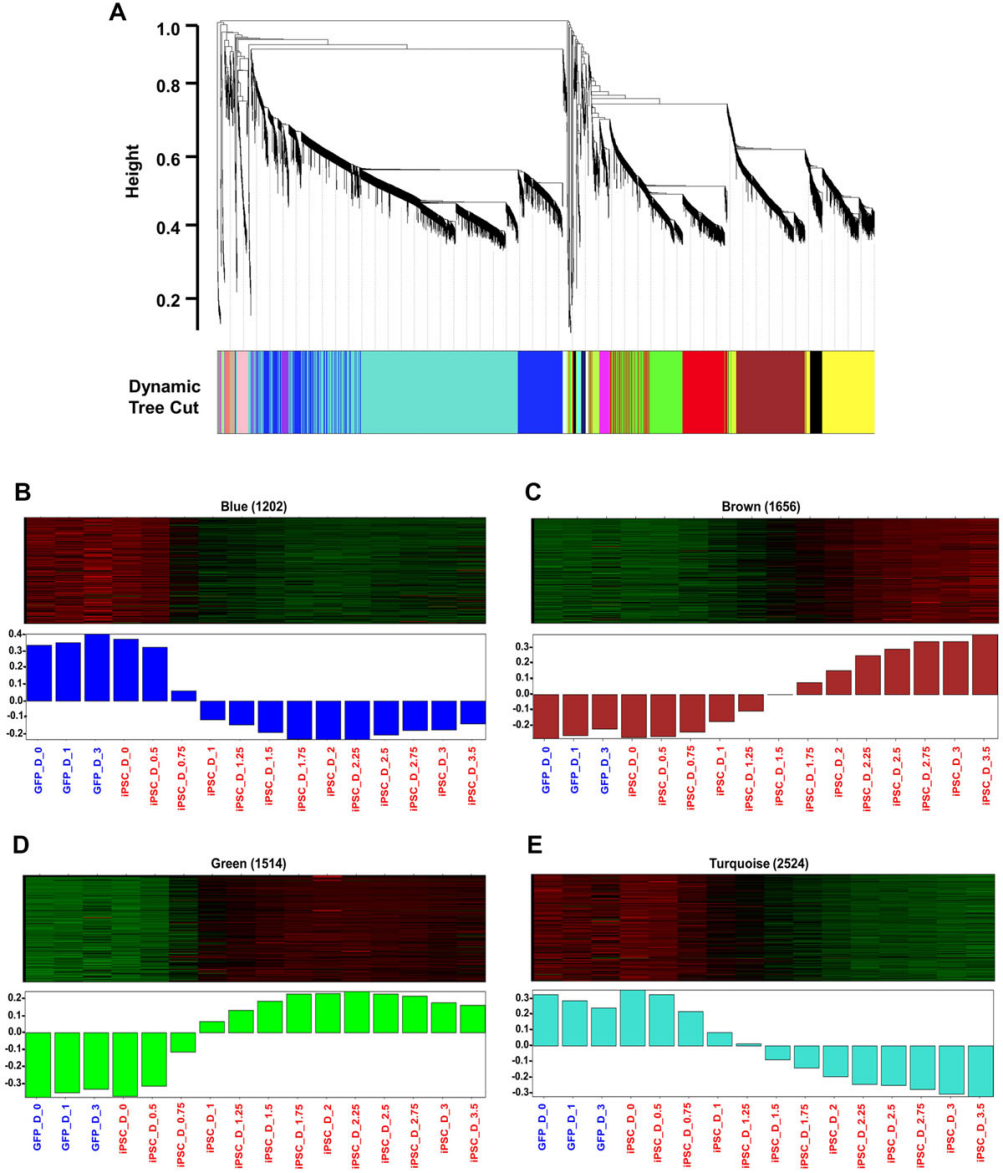
**Gene expression analyses of IMR90 cells reprogramming.** (A) Dendrogram depicting the hierarchical clustering of genes into modules based on topological overlap. Each color corresponds to a distinct module as determined by the dynamic tree cutting algorithm (Zhang and Horvath, 2005). The *y*-axis is a measure of distance determined by 1-topological overlap (see Materials and Methods). (B-E) Heat maps depicting expression levels across time points for the genes (rows) included in each module: blue (B), brown (C), green (D) and turquoise (E). Red, increased expression; green, decreased expression. Lower panel shows bar plots representing the values of the module eigengenes (i.e. the first principal component) derived for each sample from singular value decomposition for each module.

Weighted gene co-expression network analysis (WGCNA) was used to show co-expressed genes across multiple time points after reprogramming (Zhang and Horvath, 2005). Data from the two separate time series experiments shown in Fig. 4 were analyzed using WGCNA. The initial 4-time point analysis was used as validation of gene expression at the overlapping time points. The top 8000 most variable genes were used as WGCNA input and clustered based on topological overlap, a measure of shared co-expression, resulting in sixteen modules (Fig. 5A). Colors were arbitrarily assigned to each module with sizes ranging from 39 (Light-green module) to 2524 genes (Turquoise module). Modules were individually analyzed for enrichment of known iPSC markers genes, Gene Ontology (GO) terms, and KEGG pathways. Of the sixteen modules, four (Blue, Brown, Green, and Turquoise) (Fig. 5B-E) presented distinct temporal patterns of co-expression, and contained ES cell maker genes relevant to reprogramming (Yu et al., 2007; Ebert et al., 2009; Kato et al., 2007; Guo et al., 2013).

#### Blue module

The blue module contained 1202 genes that were highly expressed for the first 12 h and decreased in expression for the remainder of time series (Fig. 5B; Table S1). The blue module was enriched for genes identified as regulation of transcription (Bonferroni *P*<0.001), DNA metabolic process (Bonferroni *P*<0.001), DNA replication (Bonferroni *P*<0.001) and DNA recombination (Bonferroni *P*<0.05) by the Gene Ontology. *KLF2*, *TET1*, *TET2*, *POLI* and *MCM5* were found in this module and demonstrated low in expression across time points.

#### Brown module

The brown module was composed of 1656 genes and showed enrichment for ESC markers (*P*<0.01). Genes in this module are initially expressed at low levels, or not expressed, but start increasing expression at 48 h and continue to 84 h (Fig. 5C; Table S2).The Brown module contained the highest number of ES cell marker genes (*PODXL2*, *DNMT3L*, *ZFP42*, *CLDN6*, *PIWIL2*, *ZSCAN10*, *TERT*, *ZIC5*, *LIN28B*, *CXADR*, *NPM2*, *CTSL2*, *L1TD1*, *POU5F1* and *SALL4*). The module was enriched with genes identified as related to neurotransmitter transport (Bonferroni *P*<0.01) and neuron differentiation (*P*<0.05) by GO terms.

#### Green module

The green module contained 1514 genes that showed low expression for the first 12 h at which time expression increased continuously until 36 h (Fig. 5D; Table S3). Expression remained high across the remainder of the time points. This module was enriched for genes identified as ectoderm development (Bonferroni *P*<0.01), epidermis development (Bonferroni *P*<0.01), regulation of system process (Bonferroni *P*<0.01) by GO term. *UTF1*, *SOX15*, *NODAL*, *LEFTY1*, *LEFTY2*, *FGF4*, *KLF4* and *TFCP2L1* were included in this module.

#### Turquoise module

The turquoise module contained 2524 genes that were highly expressed at early time points with a continuous decrease in expression across the remaining time points (Fig. 5E; Table S4). The module was enriched for GO terms associated with mitosis, regulation of cell cycle and M phase, DNA repair and response to stress and DNA metabolic processes (Bonferroni *P*<0.001). Enriched KEGG terms included cell cycle, focal adhesion, meiosis, and DNA replication pathways.

## DISCUSSION

In this study, we have presented the development of an adenoviral vector harboring Yamanaka's four reprogramming factors (RFs) in a single cassette (Ad-SOcMK) under individual CMV promoters. Upon transduction of IMR90 fibroblasts with Ad-SOcMK, cells changed morphology and formed clusters as early as day 2-3. However, despite showing embryonic stem cell phenotypes, the reprogrammed cells did not show self-renewal activity. Expression array profiling during the course of reprogramming demonstrated large number of gene expression changes but some key gene expression changes are found to be opposite to those described in recent iPSC transcriptome studies. This is in contrast to true reprogramming, in which four factors trigger stochastic process where successive barriers must be overcome to reach a state toward pluripotency (Hanna et al., 2009; Yamanaka, 2009).

### Pluripotency-associated genes as demonstration of reprogramming

Somatic cells have been reprogrammed by a number of transcription factor delivery methods. It is a slow and inefficient process that requires weeks, with most cells failing to acquire a true iPSC phenotype. We overcame technical difficulties of cloning four RFs each with their own CMV promoter by reducing temperatures during clone manipulation and found that once successfully cloned into the cassette the resulting plasmid remained stable and did not recombine. Transduction of fibroblasts with the vector resulted in expression of early pluripotency markers by day two and later pluripotency markers by day three. However, we encountered the paradox that these iPSC-like cells with many of the attributes of iPSCs by mRNA or protein analysis were difficult to propagate in culture and formed teratomas inefficiently and incompletely (Figs 1–3).

### Pluripotency-associated differences are present in rapid synchronization

As the four-factor adenovirus delivery system was unable to generate reprogrammed cells with self-renewal activity, we sought to identify any perturbation of gene expression patterns linked to pluripotency from our analyses to explain the lack of acquisition and maintenance of pluripotency and cell division. As expected, the reprogramming process was accompanied by the suppression of fibroblast specific genes and activation of pluripotency-associated genes.

WGCNA allowed us to identify four key co-expression modules. Closer inspection of these modules revealed several interesting patterns. First, a subset of human ESC signature genes (*POU5F1*, *SALL4*, *UTF1*, *REX1/ZFP42*, *SOX15*, *LIN28*, *NODAL*, *LEFTY1* and *LEFTY2*) were found to be enriched in the brown and green modules (Fig. 5C,D). This was consistent with other published reports (Yu et al., 2007; Ebert et al., 2009). Downregulation of core fibroblast-specific genes (*DCN*, *COL3A1*, *GREM1*, *BDKRB1*, *LOX*, *FOXF1* and *FGF5*) in the blue and turquoise modules was evident as well (Fig. 5B,E) (Yu et al., 2007; Ebert et al., 2009). In contrast to prior studies, expression of *NANOG*, *SOX2* and *SALL2* was not increased. Some genes even showed expression changes opposite to those described in iPSCs such as increased expression of *DNMT3A* (Fig. 5C, brown module) and decreased expression of *TET1* and *TET2* (Fig. 5B, blue module). Furthermore, a large number of cell cycle and DNA replication-related genes were found to be significantly down-regulated (blue and turquoise modules in Fig. 5B,E) which is not compatible with the self-renewal nature of stem cells.

Our reprogramming assay demonstrated enrichment of human ESC signature genes (*POU5F1*, *SALL4*, *UTF1*, *REX1/ZFP42*, *SOX15*, *LIN28*, *NODAL*, *LEFTY1* and *LEFTY2*) in the brown and green modules but not *NANOG*, *FOXD3*, *SOX2* and *SALL2.* The results are similar to differentially expressed genes published by two independent groups (Yu et al., 2007; Ebert et al., 2009). Looking at genes that are differentially expressed during reprogramming, it is clear that adenoviral delivery-based reprogrammed cells are different with respect to pluripotent gene expression. Such differences might be due to lack of establishment of a complete pluripotency-associated epigenome.

### Epigenetic barrier

The immediate response to induction of reprogramming factors is resetting epigenetic reprogramming, which includes changes in DNA methylation patterns at pluripotency loci and establishment of ESC-specific gene expression (Mikkelsen et al., 2008). *DNMT3A*, *DNMT3B* (*de novo* methyltransferases) and TET enzymes are epigenetic regulators during reprogramming (Kato et al., 2007; Doege et al., 2012; Piccolo et al., 2013; Hu et al., 2014). We found increased expression of *DNMT3A* (Fig. 5C, brown module) and decreased expression of *TET1* and *TET2* (Fig. 5B, blue module) in our reprogramming assay. Depletion of either *Dnmt3a* or *Dnmt3b* facilitates reprogramming and demonstrates de-methylation of the *Nanog* promoter, while the ectopic expression of *Dnmt3a* and *Dnmt3b* significantly inhibits reprogramming (Guo et al., 2013; Pawlak and Jaenisch, 2011). TET-mediated hydroxylation is involved in epigenetic activation of silent pluripotency genes (Doege et al., 2012; Piccolo et al., 2013). Activation of DNMT3A and decreased levels of TET enzymes likely explains our failure to obtain cells with true iPSC-like characteristics.

### Cell cycle genes and pluripotency

The acquisition of a proliferative status in reprogrammed cells is greatly affected by the distinct expression patterns of cell-cycle regulatory proteins (Ruiz et al., 2011; Polo et al., 2012). The key biological functions of DNA replication and cell cycle control are unique features of high quality iPSCs. Interestingly, a large number of cell cycle and DNA replication-related genes (for example: *SKA1*, *POLI*, *RFC4* and *MCM5*) were found to be significantly downregulated (blue and turquoise modules in Fig. 5B,E) which is not compatible with the self-renewal nature of stem cells. It might be the direct result of combination of unpredicted perturbation of RFs' cooperative function and aberrant methylation patterns. The reduced expression of cell cycle genes resulted in induction of cell-cycle arrest, thereby preventing propagation of reprogrammed cells.

### PI3K/AKT signaling pathway

The PI3K/Akt pathway has been implicated in the stemness and maintenance of both human and mouse ESCs via activation of Akt1 signaling which further induces the expression of *NANOG* (Armstrong et al., 2006; Watanabe et al., 2006). Inhibition of the PI3K/Akt pathway down-regulates *NANOG* and its targets such as *TBX3* and *ESRRB*, which are key factors required for self-renewal of ESCs (Ivanova et al., 2006). Constitutive expression of *Nanog* is necessary for iPSCs maturation and self-renewal activity (Hanna et al., 2009; Silva et al., 2009; Samavarchi-Tehrani et al., 2010). Although self-renewal and proliferative genes like *FGF4* and *REX1* showed enrichment (Fig. 5C,D, brown and green modules), significant expression of *NANOG*, *TBX3* and *ESRRB* was not detected in our time course experiments. Although some demethylation of the *NANOG* promoter was seen in our reprogramming system (Fig. 3; Fig. S2), expression levels may not have been sufficient to activate target genes appropriately.

### Genetic interactions and pluripotency

The pluripotent state is controlled by vast network of genetic interactions (Chen et al., 2008; Lu et al., 2009; Van den Berg et al., 2010). A recent study has shown that sixteen genetic interactions among twelve components (*Oct4*,* Sox2*,* Nanog*,* Esrrb*,* Tfc2I1*,* Stat3*,* Gbx2*,* Klf4*,* Sall4*,* Klf2*,* Tcf3* and *MEK/ERK*) are involved in developing the pluripotency network that defines the self-renewal capabilities of pluripotent cells in the mouse ES cell model (Dunn et al., 2014). Of the twelve components, we found increased expression of *KLF4*,* SALL4*,* OCT4* and *TFCP2L1* (Fig. 5C,D, brown and green modules) and decreased expression of *KLF2* (Fig. 5B, blue module) in our reprogramming assay. Interestingly, significant expression of *NANOG*,* SOX2*,* ESRRB*,* GBX2*,* STAT3*,* TCF3* and *MEK/ERK* was not evident (Fig. 5). These results do not fit quite well with published mechanistic analyses (Dunn et al., 2014). We speculate that the lack of key genes and lack of coordinated expression patterns led to an incomplete pluripotency network.

### Conclusions

This study did not address all of the iPSC behaviors that are essential for true reprogramming. Most notably, the resulting reprogrammed cells did not show self-renewal activity which is regarded as a core element of true pluripotency as described by many published reports. The dissociation of stem-cell like phenotype and self-renewal activity is surprising. The best explanation would be destabilization of sequential reprogramming events by the overly expression of RFs. Although we showed that reprogrammed cells resembled embryonic stem cells with regard to morphology, biomarker expression, and differentiation capacity, we could not expand the colonies to assess their true stemness potential. Colonies formed more rapidly (3 to 5 days) than in many previous studies that described successful iPSC reprogramming. Despite early colony formation in our rapid synchronization was associated with large expression changes, some key gene expression changes were found to be opposite to those described in recent iPSC transcriptome studies. It is possible that the high level of synchronization constrained stochastic effects that may be necessary to generate pluripotent self-renewing iPSCs. Further studies will be required to examine whether transfection with an episomal plasmid as opposed to viral transduction could overcome the failure of reprogrammed cells to expand.

## MATERIALS AND METHODS

### Construction of adenovirus

We used the AdEasy-1 system to generate adenoviruses containing RFs in a single cassette, designated Ad-SOcMK (**S**OX2, **O**CT3/4, **c-M**YC and **K**LF4). Adenoviral plasmid (pAdEasy-1), shuttle vectors (pAdTrack or pAdTrack-CMV), competent cells (AdEasier cells: *E coli* BJ5183 containing pAdEasy-1 backbone), and packaging cells (HEK 293A) were a generous gift from Drs Coralie Poizet and Larry Kedes, University of Southern California, USA. Plasmids containing the RFs: SOX2, OCT3/4, c-MYC, and GKLF4 (pEP4 E02S ET2K, pCEP4-M2L, pEP4 E02S EN2K, and pEP4 E02S CK2M EN2L, respectively) were purchased from Addgene Inc., USA. Each of the RFs was PCR amplified from the plasmids with *Nhe*I restriction sites. The PCR products were then cloned into pre-GFP deleted pEGFP-N1 plasmid (Clontech Inc., USA) at the *Nhe*I site. The authenticity of each gene was verified by *Nhe*I restriction digestion analyses and DNA sequencing. In order to clone four RFs in a single cassette into the pAdTrack shuttle vector, each gene along with the CMV promoter and SV40-polyA sequences was consecutively sub-cloned into the shuttle vector (SOX2 cassette at *Hind*III site, OCT3/4 cassette at *EcoR*V site, c-MYC cassette at *Not*I site, and KLF4 cassette at *Sal*I site), designated as pSOcMK-AdTrack shuttle vector. The resultant recombinant shuttle vector is linearized by digesting with restriction endonuclease *Pme*I, and subsequently cotransformed into *E. coli* BJ5183 cells with the adenoviral backbone plasmid, pAdEasy-1 for homologous recombination. Specifically recombination was carried out at very low temperatures (18-30°C) to enhance efficiency. Recombinants were selected for kanamycin resistance, and recombination was confirmed by multiple restriction endonuclease analyses. Finally, the recombinant adenoviral plasmid, pSOcMK-AdEasy-1, was linearized with *Pac*I and transfected into adenovirus packaging cell line HEK 293A to generate adenoviruses, designated Ad-SOcMK. Production of recombinant adenoviruses was tracked by analysis of GFP expression.

### Cell culture

Human embryonic fibroblast IMR90 cells (#CCL-186, ATCC Inc., USA), human skeletal muscle cells (SkMCs) (# CC-2661, Lonza Inc., USA), and spinocerebellar ataxia 2 (SCA2) patient skin fibroblasts containing (# GM04319, Coriell Cell Repositories, USA) were used in this study. IMR90 cells were maintained in DMEM medium containing 10% fetal bovine serum (FBS). SkMCs were maintained in SkGM medium (# 3160, Lonza Inc., USA) containing 10% FBS, and SCA2 skin fibroblasts were cultured in MEM medium containing 15% FBS.

### Adenovirus transduction and reprogramming

IMR90 cells (1.0-1.5×10^6^) were cultured overnight on 100 mm dishes without feeder cells. On the following day, cells were transduced with Ad-SOcMK or Ad-GFP (control). Adenoviruses were removed at 24 h post-transduction (day 1), and replaced with human ESC medium consisting of DMEM/F12 (#11330-32, Invitrogen Inc., USA), 20% Knockout Serum Replacement (KSR) (#10828-028, Invitrogen Inc., USA), 1× nonessential amino acids, 1× sodium pyruvate, 1× L-glutamine, 0.1 mM β-mercaptoethanol, 25 ng/ml basic fibroblast growth factor (bFGF) (#PHG0263, Invitrogen Inc., USA), and 0.5% penicillin-streptomycin. The medium was changed every day and by days 2-3, several colonies showing ESC-like morphology emerged on the dish. Feeder cells were not used.

### Western blot analyses

Protein extracts were resolved by SDS-PAGE and transferred to Hybond P membranes (Amersham Bioscience Inc., USA). After blocking with 5% skim milk in 0.1% Tween 20/PBS, the membranes were incubated with primary antibodies in 5% skim milk in 0.1% Tween 20/PBS for 2 h at room temperature or overnight at 4°C. After several washes with 0.1% Tween 20/PBS, the membranes were incubated with the corresponding secondary antibodies conjugated with HRP in 5% skim milk in 0.1% Tween 20/PBS for 2 h at room temperature. Following three additional washes with 0.1% Tween 20/PBS, signals were detected by using the Immobilon Western Chemiluminescent HRP Substrate (#WBKLSO100, Millipore Inc., USA) according to the manufacturer's protocol. The antibodies with their sources and dilutions are listed in Table S5.

### Alkaline phosphatase staining and immunocytochemistry

Alkaline phosphatase (ALP) staining was performed using the Alkaline Phosphatase Detection Kit (#SCR004, Millipore Inc., USA). Briefly, reprogrammed cells were fixed with 4% paraformaldehyde/PBS for 2 min, followed by 15 min incubation with staining solution according to the manufacturer's protocol. For immunocytochemistry, cells were fixed in 4% paraformaldehyde/PBS for 20 min at room temperature. The cells were then permeabilized with 70% ethanol and stored at 4°C. After washing with PBS, cells were blocked with 10% BSA/PBS for 2 h at room temperature. Slides were incubated with primary antibodies in 10% BSA/PBS for 2 h at room temperature or overnight at 4°C, washed three times with PBS and incubated with the corresponding secondary antibodies conjugated with Dylight variants. Following incubation, cells were washed three times with PBS and nuclei stained with 4′,6-diamidino-2-phenylindole (DAPI) (Sigma Inc., USA). The slides were mounted with mounting medium, Vectashield (Vector Inc., USA) and cells were visualized using confocal microscopy (Nikon Eclipse Ti microscopy). The antibodies with their sources and dilutions are listed in Table S5.

### PCR analyses

Total RNA was prepared from harvested cells using the RNAeasy Kit (Qiagen Inc., USA). cDNA was synthesized from 5 μg of total RNA using MMLV reverse transcriptase and random hexanucleotide primers (New England Biolab Inc., USA) according to the manufacturer's protocol. To study gene expression, cDNAs (150 ng for semi-quantitative and 5 ng for quantitative PCR) derived from the total RNA was subjected to PCR analysis. In regular PCR, the PCR products were cloned and verified by sequencing. Quantitative RT-PCR was performed in Bio-Rad CFX96 (Bio-Rad Inc., USA) with the Power SYBR Green PCR master mix (Applied Biosystems Inc, USA). PCR reaction mixtures contained SYBR Green PCR master mix and 0.5 pmol primers and PCR amplification was carried out for 45 cycles: denaturation at 95°C for 10 s, annealing at 60°C for 10 s and extension at 72°C for 40 s. The threshold cycle for each sample was chosen from the linear range and converted to a starting quantity by interpolation from a standard curve run on the same plate for each set of primers. All gene expression levels were normalized to the *GAPDH* mRNA levels. Primer sequences used for RT-PCR and qPCR are listed in Tables S6 and S7.

### Bisulfite sequencing

To assess the methylation status of CpGs in the promoter region of *NANOG*, genomic DNA was purified from IMR90 cells transduced with Ad-GFP or Ad-SOcMK using the DNeasy Kit (Qiagen Inc., USA). Purified genomic DNA (1 μg) was used to convert unmethylated cytosines (C) to uracil (U) using EZ DNA methylation kit (#D5001, Zymo Research Inc., USA), according to the manufacturer's protocol. Treated DNA was purified with QIAquick column (Qiagen Inc., USA) and purified DNA (150 ng) from each sample was subjected to PCR analyses for the promoter region of *NANOG* using the following primers: forward 5′-CACCATGCGTGGCTAATTTTTGTA-3′, reverse 5′-TTAAAATCCTGGAGTCTCTAGATTT-3′. The resulting PCR products were subcloned into the pCR2.1-TOPO vector (Invitrogen Inc., USA). Ten clones of each sample were verified by sequencing.

### Southern blotting

For Southern blot analyses, geneomic DNA from reprogrammed cells, IMR90 cells and Ad-SOcMK plasmid DNA were digested with *Bam*HI and *Asc*I for KLF4 and c-MYC, respectively. Digested DNA fragments were separated on 0.8% agarose gels and transferred to a nylon membrane (Amersham Bioscience Inc., USA). The membranes were hybridized with radioactive probes (KLF4 and c-MYC) using standard techniques. The probes used for Southern blot analyses consists of a 415 bp (KLF4; ORF1-415) and 495 bp (c-MYC; ORF 1-495) fragments of KLF4 and c-MYC cDNA sequences shown in Figs S3 and S4.

### Karyotyping

IMR90 cells were transduced with Ad-SOcMK. As cell clumps were formed cells were transferred onto inactivated MEF cells in 25 cm^2^ flask at day 3 and cultured for additional 3 days with human ES cell medium. Cells at 70-80% confluence were mitotic arrested with 50 μl of colcemid (0.05 μg/ml) for 4 h at 37°C and harvested using 0.05% Trypsin-EDTA. Hypotonization was performed with 0.075 M KCl and a few drops of Carnoy's fixative (3:1 methanol:acetic acid) were used for cell fixation. Cell suspension was dropped onto slides and then placed in a 95°C oven for 30 min. The slides were stained using standard G-banding procedures. Metaphase cells were located using the MetaSystems scanning system and analyzed for cytogenetically visible abnormalities. Imaging and karyotyping were performed in Cytogenetics Core, ARUP laboratories at the University of Utah.

### *In vitro* differentiation

To determine the differentiation ability of reprogrammed cells *in vitro*, we used the floating culture method to form EBs. Briefly, IMR90 cells were transduced with Ad-SOcMK. On day three, the resultant cells were mechanically dissociated and cultured in ESC medium (without bFGF) in non-coated T25 flasks. The medium was changed every other day. After seven days in floating culture, ball-shaped structures typical for EBs were formed. EBs were then transferred to 0.1% gelatin-coated chamber slides using the same medium. The medium was changed every other day once EBs were attached to the slide. Differentiated cells were fixed after eight days in adherent culture and stained with antibodies recognizing marker proteins for each germ layer.

### Teratoma formation

To examine the *in vivo* development potential of iPSCs, IMR90 cells were transduced with Ad-SOcMK. On day three, the resultant reprogrammed cells were injected subcutaneously into three 6-week-old male nonobese diabetic severe combined immunodeficient (NOD/SCID) mice (Jackson Laboratory) (3×10^6^ cells for each mouse). For control experiment, IMR90 cells (3×10^6^ cells) were also injected into one mouse. After 10-11 weeks, three tumors were developed and one tumor with relatively larger size (11.5 mm×10.5 mm) was dissected and fixed in 4% paraformaldehyde. Teratoma experiments were conducted in Comparative Oncology Resource Core at the University of Utah. Paraffin embedded tissue slides were hematoxylin and eosin (H&E) stained, and immunostained in the Tissue Resource and Application Core (TRAC) and Department of Pathology at the University of Utah. All procedures were performed in accordance with protocols approved by the University of Utah Animal Research Committee guidelines.

### Microarray analyses

IMR90 cells were transduced with Ad-SOcMK or Ad-GFP. Adenoviruses were removed at 12 h post-transduction and cells were sampled at every 6 h. Total RNA was prepared from each sample using Qiagen RNeasy kit according to manufacturer's protocol. Human genome SurePrintG3 8×60K carrying 27,958 genes and 7419 LincRNA targets (Agilent Technologies, Inc.) were used for microarray hybridization to examine the global gene expression. Approximately 1 μg of RNA from each sample was labeled using Agilent Two-Color Quick Amp Labeling Kit following manufacturer's instructions. All arrays were hybridized at 65°C for 17 h and scanned using an Agilent scanner G2505C. Raw intensity data were extracted using Agilent Feature Extraction Software version 10.5. Quality control was done on the basis of Agilent quality control metrics. Array experiments were performed in the Microarray Core Facility at the University of Utah.

### Weighted gene co-expression network analyses (WGCNA)

A signed WGCNA was conducted as previously described using the R package WGCNA (Langfelder and Horvath, 2008). Briefly, correlation coefficients were constructed between expression levels of the 8000 most variable probes, and a connectivity measure (topological overlap, TO) was calculated for each gene by summing the connection strength with other genes (adjacency matrix) after correlation coefficients were raised to a power of 16. The creation of an adjacency matrix aids in discarding probe similarities with low correlation and emphasizes relationships with high correlation, creating a network with a small number of biologically relevant hub genes. The TO measure was converted to a dissimilarity measure (1-TO measure) and clustered using the flashClust function which utilizes hierarchical clustering. The cluster tree was then cut with each branch corresponding a module, identified by color. Minimum module size was set to 30 genes and modules were merged if they had at least 5% similarity. We identified sixteen modules, each summarized by its primary principal component or module eigengene, considered to be representative of the gene expression profiles in a module. The correlation to the module eigengene was used to prioritize genes in each module (kME). Gene ontology annotation was performed using DAVID (http://david.abcc.ncifcrf.gov/). Microarray data have been deposited in the NCBI Gene Expression Omnibus database (GEO, www.ncbi.nlm.nih.gov/geo, accession number: GSE72676).
